# Quantitative Structure-Activity Relationship of Humic-Like Biostimulants Derived From Agro-Industrial Byproducts and Energy Crops

**DOI:** 10.3389/fpls.2020.00581

**Published:** 2020-05-26

**Authors:** Davide Savy, Yves Brostaux, Vincenza Cozzolino, Pierre Delaplace, Patrick du Jardin, Alessandro Piccolo

**Affiliations:** ^1^Plant Sciences, Gembloux Agro-Bio Tech, University of Liège, Liège, Belgium; ^2^Statistical Modelling and Development, Gembloux Agro-Bio Tech, University of Liège, Liège, Belgium; ^3^Interdepartmental Research Centre of Nuclear Magnetic Resonance for the Environment, Agri-Food and New Materials, University of Naples Federico II, Naples, Italy; ^4^Department of Agricultural Sciences, Università di Napoli Federico II, Naples, Italy

**Keywords:** biostimulants, humic-like substance, biorefinery and agro-industrial byproducts, projection on latent structure regression, partial least square regression, solid-state ^13^C-CPMAS NMR spectroscopy, liquid-state ^31^P-NMR spectroscopy, 2-chloro-4, 4, 5, 5-tetramethyl-1, 3, 2, -dioxaphospholane

## Abstract

Humic-like substances (HLSs) isolated by alkaline oxidative hydrolysis from lignin-rich agro-industrial residues have been shown to exert biostimulant activity toward maize (*Zea mays* L.) germination and early growth. The definition of a quantitative structure-activity relationship (QSAR) between HLS and their bioactivity could be useful to predict their biological properties and tailor plant biostimulants for specific agronomic and industrial uses. Here, we created several projection on latent structure (PLS) regression by using published analytical data on the molecular composition of lignin-derived HLS obtained by both ^13^C-CPMAS-NMR spectra directly on samples and ^31^P-NMR spectra after derivatization of hydroxyl functions with a P-containing reagent (2-chloro-4,4,5,5-tetramethyl-1,3,2-dioxaphospholane). These spectral data were used to model the effect of HLS on the elongation of primary root, lateral seminal roots, total root apparatus, and coleoptile of maize. The ^13^C-CPMAS-NMR data suggested that methoxyl and aromatic moieties positively affected plant growth, while the carboxyl/esterified functions showed a negative impact on the overall seedling development. Alkyl C seems to promote Col elongation while concomitantly reducing that of the root system. Additionally, ^31^P-NMR-derived spectra revealed that the elongation of roots and Col were enhanced by the occurrence of aliphatic hydroxyl groups, and guaiacyl and *p*-Hydroxyphenyl lignin monomers. The PLS models based on raw dataset from ^13^C-CPMAS-NMR spectra explained more than 74% of the variance for the length of lateral seminal roots, total root system and coleoptile, while other parameters derived from ^13^C-CPMAS-NMR spectra, namely the Hydrophobicity and Hydrophilicity of materials were necessary to explain 83% of the variance of the primary root length. The results from ^31^P-NMR spectra explained the observed biological variance by 90, 96, 96, and 93% for the length of primary root, lateral seminal roots, total root system and coleoptile, respectively. This work shows that different NMR spectroscopy techniques can be used to build up PLS models which can predict the bioactivity of lignin-derived HLS toward early growth of maize plants. The established QSAR may also be exploited to enhance by chemical techniques the bioactive properties of HLS and enhance their plant stimulation capacity.

## Introduction

Plant biostimulants are a novel class of fertilizing products that improve the “plants’ nutrient use efficiency, tolerance to abiotic stress, quality traits, and/or increasing the availability of confined nutrients in the soil or rhizosphere” (EU Fertilising Products Regulation, 1009/2019). Among several categories of biostimulants, Humic Substances (HS, including fulvic and humic acids) and HLSs are the most relevant and intensively studied (2019). They are extracted from soils, sediments, composted biomasses or agro-industrial residues and might be regarded as supramolecular associations of small, heterogeneous molecules held together in metastable structures by non-covalent interactions (van de Waals, *π*-*π*, H-bonds; metal bridges) (Piccolo et al., 2019; Wells and Stretz, 2019). Extracting HLS from energy crops or agro-industrial residues is also important from the environmental point of view, because it involves recycling precious photosynthate, which would be otherwise burnt or landfilled (Cherubini, 2010).

Both HS and HLS are reckoned to boost crop growth and yields, and protect plants from abiotic stresses, by triggering specific metabolic routes (Aguiar et al., 2016; Khaleda et al., 2017; Vinci et al., 2018). HS and HLS have also been applied to seeds or hydroponically-grown plants to study their bioactivity without the environmental complexities of field trials (Ertani et al., 2013; Savy et al., 2018; Spaccini et al., 2019).

For example, the positive bioactivity of HLS isolated by modifying the lignin contained in several agricultural residues or biorefinery wastes have been ascertained on maize (*Zea mays* L.) germination and early growth (Savy et al., 2015a, 2016a, 2017b). Although such HLS materials were not found to significantly promote seed germination percentage, a positive dose-dependent elongation of primary and lateral seminal roots, as well as of coleoptile, was recorded. As for other HS and HLS, the biostimulation caused by the above-mentioned lignin-derived HLS was related to their content of phenolic molecules and their role in affecting specific plant hormonal balances was suggested (Ray, 1986; Savy et al., 2017a). An important objective of the biostimulants industry is to understand the mode of action of a product allowing to predict its biological effect toward plant growth. In order to reach this aim, a QSAR should be derived. QSAR is a mathematical equation relating the biological properties of a material to some of its chemical and physical characteristics (Roy et al., 2015). Once a model for HLS bioactivity is defined, the expected bioactivity of other biostimulants from lignin-rich sources could be predicted. Furthermore, the production of humic-like biostimulants with the desired biological properties may be based on the selected QSAR model, thus reducing the risk of applying HLS with low bioactivity.

In order to optimize a QSAR model for a specific bioactive material, its molecular characterization should be as detailed as possible (Roy et al., 2015). Despite the efforts to define the molecular composition of the heterogeneous HS and HLS, unraveling their complex structure still remains a challenge (Nebbioso and Piccolo, 2011; Nebbioso et al., 2014; Drosos et al., 2017, 2018). Nevertheless, QSAR for HS had been obtained based only on NMR spectra of the bulk humic matter (Geyer et al., 1998; Steinberg et al., 2000; Aguiar et al., 2013). However, misleading interpretations may arise if linear regressions and multiple linear regressions are used to build the models, since they assume *non*-correlated predictors, whereas the HS and HLS chemical data used as predictors are expected to be correlated with each other. Conversely, Principal Component Regression and PLS (also known as Partial Least Square) regression provide accurate QSAR models even when dealing with highly correlated independent variables (Esbensen, 2001), and had been successfully applied to derive QSAR for HS from different sources (Canellas et al., 2012; García et al., 2016). Although both statistical tools are valid for developing prediction models, PLS regression is a more efficient technique than Principal Component Regression. In fact, the PLS regression sequential extraction of model components is a one-step process, and it is carried out by using both chemical and biological data simultaneously, whereas the Principal Component Regression extracts such components in a two-step process. Moreover, PLS regression usually requires fewer components than Principal Component Regression to achieve the same prediction level.

In this work, we derived a QSAR for HLS extracted from lignin-rich agricultural and biorefinery residues, by applying PLS regression to a dataset retrieved from previously published articles, in which both the chemical structure and the biological activity of such plant enhancers were reported (Savy and Piccolo, 2014; Savy et al., 2015a, 2016a, 2017b). In particular, HLS were chemically characterized by both solid-state ^13^C-CPMAS and liquid-state ^31^P NMR spectroscopy, whereas the HLS bioactivity was evaluated toward maize germination and early growth. Hence, our aims were: (i) to create two PLS models by relating spectral data from either ^13^C-CPMAS-NMR or ^31^P-NMR spectra of selected HLS to their bioactivity; (ii) to assess the accuracy of the achievable prediction by using the two models and (iii) to identify the most important HLS chemical features that exert the bioactivity on plants.

## Materials and Methods

### Plant Biomasses

Cardoon (*Cynara cardunculus* L) was cropped on September 2012 at the University of Naples experimental farm in Bellizzi (Salerno, Italy), while eucalypt (*Eucalyptus camaldulensis* Dehnh.) was harvested on March 2012 at an experimental farm near Eboli (Italy). Black poplar (*Populus nigra* L.) was cropped on March 2012 from along either the Ripiti (Salerno, Italy) or the Limatola (Benevento, Italy) creeks. Miscanthus (*Miscanthus* x *giganteus* Greef et Deuter) was provided by Phytatec, Ltd. (United Kingdom), after being harvested on February 2007 in Aberystwyth (United Kingdom), whereas giant reed (*Arundo donax* L.) was cropped on January 2010 at the experimental farm of the University of Naples Federico II near Salerno (Italy). All the above-mentioned biomasses were selected because of their relevance in the production of either paper or energy (Benjelloun-Mlayah et al., 1997; Christersson, 2008; Cotana et al., 2015; Ge et al., 2016; Vilasboa et al., 2019). Finally, two biorefinery residues were obtained by hydrolyzing giant reed biomass to produce succinic acid (Savy et al., 2017b) and the hydrolyzed solid residues were then subjected to HLS extraction.

### Extraction of Humic-Like Substances (HLS) by Alkaline Oxidative Hydrolysis

The HLS were isolated according to Sun et al. (2000). Briefly, an alkaline 2% H_2_O_2_ (v/v) aqueous solution (pH 11.6) was added to the lignocellulosic substrates and stirred overnight at 323 K. The mixture was then centrifuged (15,400 RCF x 20 min) and the supernatant dialyzed (1 kDa cut-off dialysis tubes) against deionized water, freeze-dried, and stored in dried conditions for further analyses. The amount of inorganic compounds in the HLS was previously showed to be negligible, i.e., the biostimulant effect observed is conceivably related only to the organic molecules they contained (Savy and Piccolo, 2014; Savy et al., 2015a, 2017b). HLS obtained from cardoon, eucalypt, poplars from Ripiti and Limatola, miscanthus, giant reed and the two biorefinery wastes will be referred to as CAR, EUC, RIP, LIM, MG, AD, BYP 1, and BYP 2, respectively.

### Synthesis of 2-Chloro-4,4,5,5-Tetramethyldioxaphospholane and HLS Derivatization Prior to ^31^P-NMR Spectroscopy

The derivatizing phosphorous reagent 2-chloro-4,4,5,5-tetramethyldioxaphospholane (TDMP) was synthesized as described in Savy et al. (2017b). Briefly, TDMP was obtained by mixing the two following solutions: solution (A), prepared by dissolving 21.5 mL PCl_3_ in 180 mL of dry n-hexane placed in a 250 mL three-necked round flask equipped with a condenser, and solution (B), prepared by dissolving 23.7 g pinacol in a mixture of 32 mL of dry pyridine and 150 mL of dry n-hexane placed in a conic flask. Solution (B) was added drop-wise to solution (A) using an addition funnel placed on the second neck of the round flask. The addition lasted 1 h under vigorous stirring in an ice bath, then and the mixture was left for 1 h at room temperature to complete the reaction. The solution was filtered on a filter paper, while the whitish residue on the filter was rinsed with 2 × 100 mL of n-hexane and the filtrates were evaporated under *vacuum* at 328 K. Finally, CTMP was separated from solution by *vacuum* distillation (b. p. 370 K at 4 mbar).

The hydroxyl (OH) groups in the HLS were derivatized with TDMP as it follows. A stock solution was prepared by adding 2.92 mg mL^–1^ cyclohexanol (used as internal standard), 10.0 mg mL^–1^ of triphenyl phosphate (as reference peak for the ^31^P frequency axis calibration) and 0.6 mg mL^–1^ of chromium (III) acetylacetonate (as relaxation agent) to a pyridine and deuterated chloroform solution (1.6/1 v/v). The HLS (7.0 mg) were the dissolved in 750 μL of the stock solution and added with 50 μL of TDMP. All HLS were fully soluble in the solvent mixture used.

### Nuclear Magnetic Resonance (NMR) Spectroscopy

The solid-state CPMAS NMR spectra were acquired with a 300 MHz Bruker Avance magnet (Bruker Bio Spin GmbH, Rheinstetten, Germany), composed of a wide-bore system and equipped with a CPMAS (Cross-Polarization Magic-Angle-Spinning) probe, working at ^13^C frequency of 75.47 MHz. Samples were loaded into 4-mm zirconia rotors, closed with Kel-F caps and spun at a rate of 10000 ± 1 Hz. Such spectra were acquired by applying a cross-polarization technique and consisted of 1814 time domain points, a spectral width of 300 ppm (22,727.3 Hz), a recycle delay of 2 s, 5000 scans and 1 ms of contact time. The ^13^C-CPMAS pulse sequence was conducted by using a ^1^H Ramp pulse to account for the *non-*homogeneity of the Hartmann–Hahn condition. A TPPM15 scheme was applied to perform the ^13^C-^1^H decoupling. The free induction decay (FID) was transformed by applying a 4k zero filling and an exponential filter function with a line broadening of 100 Hz.

The ^31^P-NMR spectra were obtained with a 400 MHz Bruker Avance spectrometer (Bruker Biospin, Rheinstetten, Germany), equipped with a 5 mm Bruker Inverse Broad Band (BBI) probe, working at ^1^H and ^13^C frequencies of 400.13 and 100.62. Such spectra were acquired on TDMP-derivatized HLS by applying an inverse gated pulse sequence including a 80 μs length (15.6 dB power level) Waltz16 scheme to decouple phosphorous from proton nuclei. In particular, spectra consisted in a 45° pulse length of 5.25 μs, a spectral width of 400 ppm (64,935.066 Hz), 10 s of recycle delay, 1600 transients, 8 dummy scans and 129,862 time domain points.

All NMR spectra were acquired at a temperature of 298 ± 1 K and processed by using either Bruker Topspin Software (v.2.1, Bruker Biospin, Rheinstetten, Germany) or MestReC NMR Processing Software (v.4.8.6.0, Cambridgesoft, Cambridge, MA, United States). Zero filling was applied during Fourier transform of FIDs.

### Germination of Maize Seeds and Seedling Emergence

Maize (*Zea mays* L.) seeds were soaked in tap water overnight and fifteen (15) seeds were deposited for each replicate on round filter paper placed in a Petri dish. At least three replicates were used per each experiment. The filters were moistened with aqueous solution of HLS at various concentrations (ranging from 0 to 100 mg HLS L^–1^) and the seeds were germinated in the dark at 298 K for 96 h. Thereafter, the plantlets were scanned with a modified flatbed scanner (Epson Perfection V700, Seiko Epson, Corp., Japan) and the length of primary root, lateral seminal roots, total root apparatus and coleoptile was measured by using the WinRhizo Pro software, version 2016 (Regent Instruments, Inc., Canada).

### Projection on Latent Structure (PLS) Regression

Prior to run the PLS regression, the biological data were normalized with respect to the control mean for each specific experiment, which was set to 100. Furthermore, the models were built by selecting the treatment means that in most cases provided the longest value for the length of primary root, lateral seminal roots, total root system or coleoptile (Savy et al., 2015a, 2016a, 2017b). The dataset consisted of a matrix (8 × 20) with the HLS in columns and spectral data or biological parameters in rows. Specifically, 10 rows corresponded to the ^13^C-CPMAS-derived data, 6 rows contained the ^31^P-derived NMR data, and 4 rows showed the HLS-elicited biological response. The chemical information obtained by either ^13^C-CPMAS- or ^31^P-NMR spectra were employed as predictors of the biological results to build up two different PLS models per each biological parameter. Since the data from both the NMR techniques employed could not be always obtained due to technical and budget limitations, two different PLS models were created by using results from each NMR spectroscopic analysis, instead of building one only PLS model by combining results from both techniques. Hence, if the models sufficiently explain the variance of our biological data, their predictive power for future applications may be valid even though only one of the spectral techniques is available. The optimum number of latent factors was calculated by leave-one-out cross-validation. The latent variables (or factors) are variables that can capture an underlying phenomenon being investigated and, since cannot be directly measured, are calculated from the actual measurements; hence they are correlated with them. Also, the exploitation of latent variables represents a convenient mean to summarize the observed (X) variables by using much fewer factors (Bollen, 2002). All the statistical analyses were performed by using OriginPro, “2017” Version (OriginLab Corporation, Northampton, MA, United States).

## Results

### Chemical Features and Bioactivity of the Different HLS

The molecular composition based on NMR spectra of various HLS and their bioactive responses on maize germination are reported in Table 1. Except for MG and AD, the HLS had generally large relative alkyl contents, with EUC showing the greatest value (38.0%). Conversely, both AD and MG contained larger methoxyl groups than the other HLS, with the exception of EUC, whose methoxyl amount was comparable to that of AD (Table 1). The *O*-alkyl groups, usually associated with the resonance of carbons in lignin lateral chains and carbohydrates, were larger in CAR- and LIM-derived materials (Robert, 1992; Spaccini et al., 2019). Furthermore, the occurrence of carbohydrates is confirmed by NMR signals in the 90–110 ppm range, attributed to the resonance of anomeric carbons (Table 1). The aromatic and phenolic content in HLS isolated from Liliopsida (MG, AD, BYP 1, and BYP 2) was more abundant than in those extracted from Magnoliopsida (CAR, RIP, LIM, and EUC) (Table 1). Finally, the relative amount of carboxyl/esterified C was larger in the two poplar-derived HLS, followed by EUC, BYP 2 and MG, which showed comparable amount of such units, and then by BYP 1, CAR and AD (Table 1).

**TABLE 1:** Carbon compounds and OH functional groups observed by ^13^C-CPMAS- and ^31^P-NMR spectra, respectively, for different lignin-derived HLS, and their bioactive responses toward maize germination.

	**Peak attribution (chemical shift-ppm)**	**MG**	**AD**	**BYP 1**	**BYP 2**	**CAR**	**RIP**	**LIM**	**EUC**
**^13^C-CPMAS-NMR**	**Alkyl C (0-45)**	9.3	5.7	19.7	17.4	17.1	25.0	18.9	38.0
	**Methoxyl C (45-60)**	22.1	17.7	16.0	15.6	14.1	14.8	14.9	18.6
	***O*-alkyl C (60-90)**	24.0	24.6	17.9	20.5	33.9	25.2	29.1	19.4
	**Anomeric C (90-110)**	10.9	12.5	9.4	8.6	11.1	8.9	10.1	7.0
	**Aryl C (110-145)**	21.5	27.3	24.6	24.5	15.7	14.4	13.5	9.0
	***O*-aryl C (145-160)**	6.8	9.4	7.7	7.6	4.3	4.6	6.5	2.0
	**Carboxyl/esterified C (160-190)**	5.5	2.8	4.7	5.8	3.8	7.1	7.0	6.0
	**Hydrophobicity**	37.6	42.4	52.0	49.4	37.1	44.0	38.9	49.0
	**Hydrophilicity**	62.4	57.6	48.0	50.6	62.9	56.0	61.1	51.0

**^31^P-NMR**	**Aliphatic OH groups (150.8-146.3)**	5.5	5.7	1.6	1.6	6.8	5.4	4.7	3.9
	**Syringyl groups (143.3-142.2)**	0.07	0.18	0.04	0.06	0.14	0.07	0.11	0.04
	**Condensed phenolic groups (142.8-141.7)**	ND	ND	0.07	0.10	0.14	0.04	0.04	0.11
	**Guaiacyl groups (140.2-138.4)**	0.46	0.63	0.28	0.30	0.11	0.07	0.14	0.04
	***p*-Hydroxyphenyl groups (138.6-136.9)**	0.28	0.18	0.13	0.15	ND	0.04	0.07	0.07
	**Carboxyl OH groups (135.6-133.7)**	0.9	1.0	0.9	0.7	0.4	1.0	1.2	1.4

**Biological variables**	**Primary root length**	141.2	149.7	115.0	109.7	107.7	116.4	98.4	88.0
	**Lateral seminal root length**	174.1	153.1	111.7	141.1	140.7	120.7	106.9	80.8
	**Total root length**	156.3	151.5	113.0	127.0	123.0	119.0	103.0	83.3
	**Coleoptile length**	146.5	131.9	105.1	113.6	175.0	109.8	103.8	95.3

*The ^13^C-CPMAS spectral data are expressed as percent of total spectral area, while ^31^P-NMR data are reported as mmol OH g^–1^ of HLS. The biological results refer to raw data normalized with respect to the control mean, which was set to 100. Hydrophobicity = sum of signal areas corresponding to the resonance of alkyl, aryl and *O*-aryl C; Hydrophilicity = sum of signal areas corresponding to the resonance of methoxyl, *O*-alkyl, anomeric and carboxyl C. ND = Not Detected.*

Useful indicators of the hydrophobic or hydrophilic character of substrates are calculated from ^13^C-CPMAS-NMR spectra as Hydrophobicity and Hydrophilicity (Table 1). In fact, Hydrophobicity corresponds to the sum of areas under the signals of alkyl, aryl and *O*-aryl C, while Hydrophilicity refers to the sum of signals areas for methoxyl, *O*-alkyl, anomeric and carboxyl C (Canellas et al., 2012). BYP 1, BYP 2, and EUC showed a larger Hydrophobicity than other HLS, mainly due to the significantly larger relative content of alkyl groups. The largest hydrophilic character of the remaining HLS can be mainly ascribed to the large relative amount of methoxyl, *O*-alkyl and anomeric C (Table 1).

The ^31^P-NMR spectra of HLS previously derivatized with TMDP are used to quantitatively assess the amount of different hydroxyl (OH) functional groups present in HLS, namely in aliphatic, carboxyl and phenolic components (Granata and Argyropoulos, 1995). Besides allowing the qualitative and quantitative appraisal of the OH moieties, ^31^P-derivatizaion of HLS significantly enhanced the spreading of NMR signals over a large width of ^31^P-NMR spectra, with consequent reduction of signal overlapping (Savy et al., 2015b, c). All these characteristics highlight the advantages of this technique to thoroughly characterize HLS-contained OH groups.

The largest amount of aliphatic OH was found in CAR, followed by MG, AD and RIP, which showed comparable amount of these functions, followed in the order by LIM and EUC. The OH aliphatic content in both materials from biorefinery residues was instead markedly smaller than for the rest of HLS (Table 1). EUC and LIM contained similar COOH amount, whereas CAR showed the smallest amount of carboxyl OH functions. The content of COOH groups for the other HLS was intermediate between those shown by LIM and CAR (Table 1).

One of the main advantages of the ^31^P-NMR technique is to enable both a qualitative and quantitative evaluation of different types of phenols contained in HLS (Granata and Argyropoulos, 1995; Savy et al., 2016b). In fact, it is possible to accurately estimate the content of lignin monomers, as well as that of condensed phenolic groups, thus providing a detailed molecular description of phenol-rich HLS. Lignin monomers commonly found in grasses are composed of guaiacyl, syringyl and *p*-hydroxyphenyl radicals (Supporting Figure SF1). Conversely, hardwood lignin is virtually composed only by guaiacyl and syringyl units (Monda et al., 2018). In fact, *p*-hydroxyphenyl-derived compounds found in EUC, LIM, RIP, and CAR were either present in very low concentration or even absent, whereas they were well-represented in HLS from grasses (Table 1). The amount of syringyl moieties was markedly larger in AD, followed by CAR and LIM, while the remaining HLS showed comparable syringyl content. Similar amount of condensed phenolic units were found in CAR, EUC and BYP 2, whereas their content was smaller in LIM and RIP and completely absent in AD and MG (Table 1). Conversely, the two latter HLS showed the largest amount of guaiacyl, followed by the two BYP materials and by the hardwood-extracted substances.

A HLS-dependent activity toward the elongation of both the root apparatus and the coleoptile was noted (Table 1). The largest primary root and total root length was promoted by addition of AD and MG, while BYP 1, BYP 2, CAR, and RIP elicited similar primary root elongation. Instead, the primary root growth induced by both LIM and EUC was similar to the control (Table 1). The development of lateral seminal roots was more pronounced for MG and AD, followed by BYP 2, CAR, BYP 1, RIP, LIM, and EUC (Table 1). Finally, CAR favored the largest coleoptile length, followed by MG and AD, whereas BYP 1, BYP 2, RIP, and LIM had the least effect. As for the other parameters, EUC addition did not significantly affected plant development compared to the control (Table 1) (Savy et al., 2015a).

### Projection on Latent Structure (PLS) Regression

The optimal number of latent factors1 extracted using data from ^13^C-CPMAS spectra was 3 for primary root elongation, 1 for both lateral seminal roots and total root length and 5 for coleoptile elongation, whereas the optimal number of latent components was 3 for primary root growth, 4 for the elongation of the lateral seminal roots and the total root system and 2 for coleoptile growth when the HLS bioactivity was predicted on the basis of ^31^P spectra (Table 2).

**TABLE 2:** Number of latent factors, and percentage of the explained cumulative variance for both predictors (VarXcum) and dependent variables (VarYcum) related to ^13^C-CPMAS-NMR and ^31^P-NMR spectral data.

		**Primary root length**	**Lateral seminal root length**	**Total root length**	**Coleoptile length**
**^13^C-CPMAS-NMR**	n° latent factors	3	1	1	5
	VarXcum	98.83	72.81	72.93	99.97
	VarYcum	83.24	74.68	81.51	98.89

**^31^P-NMR**	n° latent factors	3	4	4	2
	VarXcum	99.9	99.98	99.98	98.6
	VarYcum	89.63	95.8	96.43	92.74

**FIGURE 1:**
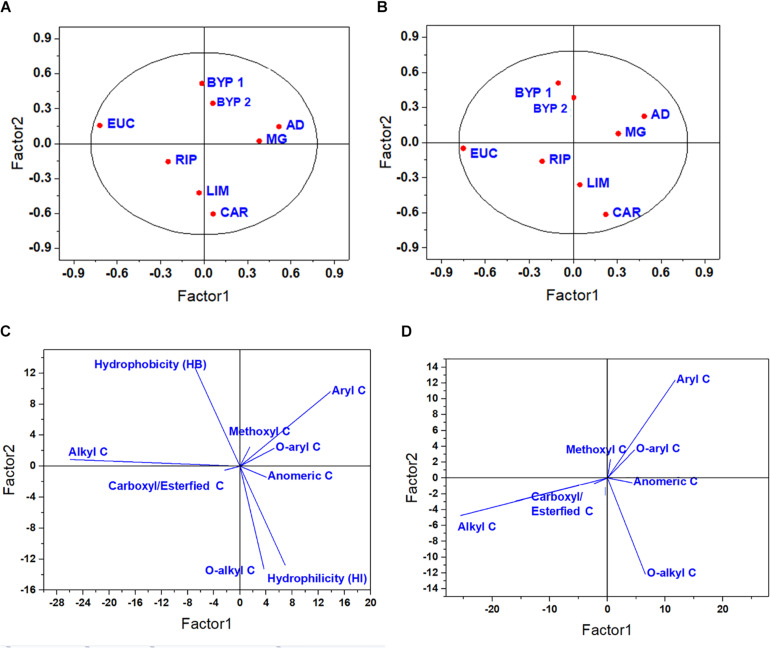
Score plot **(A,B)** and loading plot **(C,D)** for the first two latent components for primary root **(A,C)** and coleoptile **(B,D)** elongation, as related to ^13^C-CPMAS-NMR spectral data.

The score and loading plots for the first two optimal latent factors extracted from the predictor variables related to ^13^C-CPMAS-NMR spectra are reported in Figure 1. Since only one latent component was considered as optimal in the case of both LSR and TRL, only score plots and loading plots for primary root and Col are reported. In the case of primary root elongation, the HLS isolated from miscanthus, and raw or treated giant reeds were placed in the upper right-hand quadrant. Here, two clusters may be noticed: one is composed from the HLS derived from biorefinery residues, whereas the other is formed by the HLS from miscanthus and giant reed. These four HLS were placed in the same quadrant due, to their large amount of lignin-related moieties Instead, the HLS from other sources spread across the other quadrants (Figure 1A). LIM and CAR were grouped together due to their HI and large content of *O*-alkyl C, while RIP was associated with carboxyl/esterified C, and EUC did not cluster with any HLS, showing the largest alkyl C content (Figure 1C). An even clearer separation was achieved in the case of coleoptile growth. For this biological variable, the second latent factor discriminated between HLS isolated from Magnoliopsida and Liliopsida, due to their similar relative amount of lignin-related moieties, such as methoxyl, aryl and *O*-aryl C (Figures 1B,D).

**FIGURE 2:**
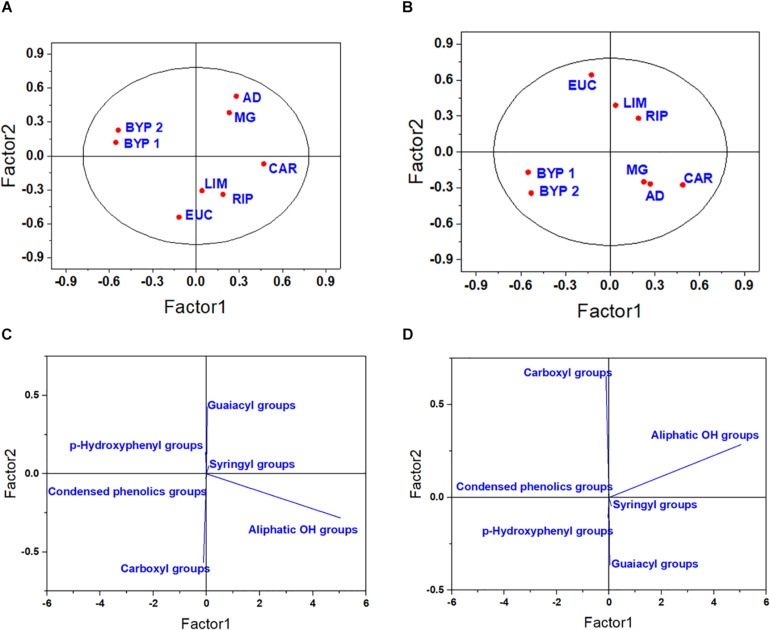
Score plot **(A,B)** and loading plot **(C,D)** for the first two latent components for the elongation of primary root **(A,C)**, and lateral seminal root **(B,D)**, as related to ^31^P-NMR spectral data.

The PLS model calculated by using the ^31^P-NMR spectra also resulted in the formation of several clusters (Figure 2). The primary root -related score plot showed that the first latent factor clustered BYP 1 and 2 together, while the second component enabled the discrimination between HLS obtained from herbaceous Angiosperms (MG, AD, BYP 1, and BYP 2) from those isolated from hardwood (CAR, EUC, RIP, and LIM) (Figures 2A,C). Since the score and loading plots calculated for the elongation of lateral seminal roots, total root system and coleoptile were all similar, only those obtained for development of lateral seminal roots are reported (Figures 2B,D), while plots for both total root system and coleoptile length are shown in Supporting Figure SF2. The score plots for LSR showed that BYP 1 and 2 were separated from the other products, and CAR tended to cluster with MG and AD, instead ofbeing grouped with other Angiosperm-derived HLS (Figures 2B,D).

Despite our models relied on a relatively limited number of HLS, the residuals for all the biological variables were found to be randomly distributed around zero, no matter of the predictor employed (not shown). This indicates that there is no drift in the process and that the model is reliable. Moreover, the PLS models calculated by using either ^13^C-CPMAS- or ^31^P-NMR spectra provided satisfactory predictions for all evaluated biological traits (Table 2). The explained cumulative variability for both predictors and biological parameters based on ^13^C-CPMAS-NMR spectra was larger than 70%, and reached almost 100% when modeling Col length. Conversely, the explained cumulative variability for the predictors and the biological parameters were always larger than 89% and even reached 100% when HLS bioactivity model was built by employing data from ^31^P spectra (Table 2). Since the varYcum indicates the extent of biological variability explained by the proposed model, it also corresponds to the coefficient of determination obtained by plotting the measured biological variables versus the predicted values (Supporting Figures SF5, SF6).

**FIGURE 3:**
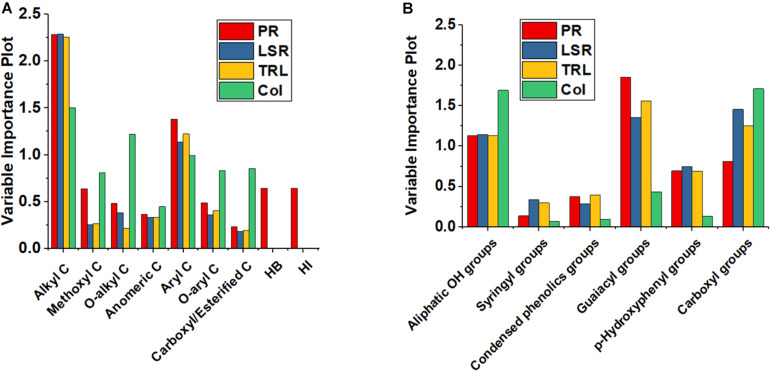
Variable Importance Plot based on ^13^C-CPMAS-NMR **(A)** and ^31^P-NMR **(B)** results.

In order to ascertain the statistical significance of each independent variable with respect to its effect on the generated model, the VIP was reported in Figure 3. The VIP describes the extent by which a model relies on each predictor, and indicates the contribution of each X variable in predicting the independent variables. The variables with larger values contribute to the model more than those with smaller values, thus entailing a greater predictive power. The most useful variables for all biological parameters when modeled on ^13^C-CPMAS-NMR spectral data are alkyl C and aryl C (Figure 3A). Furthermore, Hydrophobicity and Hydrophilicity, as well as methoxyl C had a large impact on length of primary root. Methoxyl C, together with *O*-alkyl, *O*-aryl and carboxyl/esterified C showed an important effect on coleoptile length (Figure 3A). The guaiacyl and *p*-hydroxyphenyl compounds, together with the aliphatic OH and carboxyl groups were among the most important predictors for the development of the root apparatus (Figure 3B). Instead, coleoptile development was mainly affected by OH groups in aliphatic and carboxyl moieties. Smaller effects were recorded for syringyl and condensed phenols (Figure 3B).

Although VIP is essential to unravel how well each descriptor predicts biological parameters, it does not inform on whether such predictors may positively or negatively affect the dependent variables. In order to understand the effect of the X variables toward the Y variables, the calculated regression coefficients should be considered. When basing ^13^C-CPMAS spectral data to run the PLS model, positive regression coefficients were obtained for the methoxyl and aryl C for both the root system (primary root, lateral root, total root) and coleoptile (Table 3). Also, length of lateral seminal roots, total root system and coleoptile showed positive coefficients for the *O*-alkyl groups, while a negative coefficient was calculated for length of primary root. As for the *O*-aryl C, a positive coefficient was derived for length of lateral seminal roots and total root system, while negative ones were found for coleoptile and primary root elongation, the latter being anyhow almost negligible (Table 3). In the case of anomeric C, positive coefficients were found for the root-related parameters, whereas a negative one was calculated for coleoptile. Moreover, the coefficients for alkyl groups were negative for length of primary root lateral seminal roots and total root system, whereas it was positive for coleoptile. Carboxyl/esterified C showed negative coefficients for all the evaluated biological traits evaluated (Table 3). Finally, the same but opposite coefficients were obtained for both Hydrophobicity and Hydrophilicity, being Hydrophobicity negative and Hydrophilicity positive (Table 3). It is noteworthy that Hydrophobicity and Hydrophilicity were used as predictors for length of primary root, while they were not applied for other models, since their exclusion provided larger values of the explained cumulative variability for both predictors and biological parameters (data not shown).

**TABLE 3:** Regression coefficients from PLS regression for different biological variables.

		**Primary root length**	**Lateral seminal root length**	**Total root length**	**Coleoptile length**
**^13^C-CPMAS-derived predictors**	**Alkyl C**	**−**1.17	**−**1.91	**−**1.59	0.32
	**Methoxyl C**	1.50	0.21	0.19	4.09
	***O*-alkyl C**	**−**1.07	0.32	0.15	6.78
	**Anomeric C**	0.32	0.28	0.23	**−**4.09
	**Aryl C**	0.71	0.95	0.86	6.67
	***O*-aryl C**	**−**0.03	0.30	0.29	**−**11.92
	**Carboxyl/Esterified C**	**−**0.26	**−**0.15	**−**0.14	**−**1.84
	Hydrophobicity*	**−**0.49			
	Hydrophilicity*	0.49			
	**Intercept**	117.85	131.77	125.58	**−**119.40

**31P-derived predictors**	**Aliphatic OH groups**	2.92	6.31	4.45	8.10
	**Syringyl groups**	4.27	**−**71.15	**−**49.26	2.33
	**Condensed phenolic groups**	**−**17.21	**−**62.79	**−**71.18	3.26
	**Guaiacyl groups**	74.27	34.76	49.25	15.56
	***p*-Hydroxyphenyl groups**	28.57	154.27	94.04	4.59
	**Carboxyl OH groups**	**−**8.41	**−**59.53	**−**37.35	**−**61.32
	**Intercept**	89.53	141.33	123.55	140.52

*The regression coefficient for both Hydrophobicity and Hydrophilicity were used as predictors only to model the primary root length.

The ^31^P-NMR results for TMDP-derivatized HLS suggested a positive role of aliphatic OH, and G and P groups on the elongation of all the studied plant organs, while negative coefficients were derived for carboxyl moieties (Table 3). Furthermore, the PLS model calculated positive coefficients for syringyl functionalities in the case of primary root and coleoptile elongation, while negative coefficients were observed for the length of both lateral seminal root and total root system. Finally, negative coefficients were derived for condensed phenols with respect to root-related measurements, whereas positive coefficients were calculated for coleoptile (Table 3).

## Discussion

### Pros and Cons of ^13^C-CPMAS- or ^31^P-NMR Spectroscopy for QSAR Derivation

Several models were created here to obtain a QSAR between HLS extracted by lignin-rich agro-industrial residues and their biological effect on maize early development. In the PLS model, ^13^C-CPMAS-NMR or ^31^P-NMR spectra were used as predictor variables, which enabled explanation of a large percentage of variance for all modeled biological parameters, thus indicating that these physical-chemical techniques provide an accurate prediction of HLS bioactivity (Table 2).

The explained cumulative variance based on ^31^P-NMR spectra appeared to better relate predictors and dependent variables than the ^13^C-CPMAS technique did, and allowed to acquire detailed and quantitative information on the most reactive groups of HLS. However, both methods have advantages and disadvantages. The derivatization technique applied to obtain ^31^P-NMR spectra encompasses several drawbacks. In fact, routine ^31^P analyses by this method may be difficult and costly, since the TMDP derivatizing reagent is not widely commercially available and it is rather expensive (Meng et al., 2019). Moreover, although TMDP can be synthetized in the laboratory, this is achieved through a complex procedure, and hazardous and/or deuterated solvents (pyridine and chloroform) are still required for ^31^P spectral acquisitions (Hatzakis and Dais, 2008). On the other hand, the ^13^C-CPMAS technique can be applied on samples without any preliminary treatment, but it requires a specific NMR probe for solid-state experiments, and that is not always available in routine NMR laboratories. Moreover, the solid-state technique provides only semi-quantitative data, thereby limiting its reliability in the complete characterization of a material. In spite of these drawbacks, such analytical tool was already successfully exploited to derive QSARs for HS (Canellas et al., 2012; Aguiar et al., 2013; García et al., 2016).

Despite the drawbacks showed by ^13^C-CPMAS- and ^31^P-NMR, the spectral results obtained by each technique have helped in shedding light on the molecular composition of HLS responsible for different biological activities (Table 1). The structural dissimilarities among HLS should be attributed to the different molecular nature of the original lignocellulosic biomasses and their different reactivity during the HLS extraction. For example, *p*-hydroxyphenyl moieties were not found in CAR, since cardoon lignin is composed only by syringyl and guaiacyl units (Lourençao et al., 2015), whereas lignin in giant reed, miscanthus, eucalypt and poplar contain all three monolignols, although their content vary significantly among these materials (Faix et al., 1989; Fukushima and Terashima, 1990; Evtuguin et al., 2001). Furthermore, though the AD and the BYP substrates were ultimately isolated from giant reed biomass, several discrepancies in composition were observed between the latter and the two former lignocellulosic extracts. For example, the relative content of alkyl and carboxyl C was larger in BYP1 and 2, whereas aliphatic OH functions and syringyl monolignol were larger in AD (Table 1). These differences should be attributed to the biorefinery treatments applied on giant reed, which first underwent a steam-explosion pre-treatment and then two different and enzymatic hydrolyzes, providing the two BYP products (Garbero et al., 2010; De Bari et al., 2013; Cimini et al., 2016). Hence, the hydrolytic procedures should have modified the bioactivity of the giant reed substrate, by making both aliphatic- and phenolic-containing molecules more prone to degradation, thus explaining their significantly lower amount in BYP 1 and 2 (Savy et al., 2017b).

### Different Chemical Functionalities Display Diverse Effects Toward Plant Growth

In line with previous findings, our models highlighted the dependence of HLS bioactivity upon specific chemical groups and functions (Figure 3 and Table 3). The positive role of lignin-derived aryl moieties was already reported by Canellas et al. (2012) and Aguiar et al. (2013). These authors additionally indicated the negative impact on lateral root formation of both alkyl C and carbohydrates groups present in the humic acids applied to maize. We observed positive regression coefficients for the *O*-alkyl functions that were used to model the elongation of lateral seminal roots, and the total root and coleoptile length. Our findings agree with results found by García et al. (2016), who studied the structure-activity relationship of both HS and humic acids by Principal Component Regression, and ascribed the elongation of smaller rice (*Oryza sativa* L.) roots to HS-contained labile and more functionalized structures, such as *O*- and *N*-alkyl chains.

A negative regression coefficient was found here for the Hydrophobicity, in agreement with Canellas et al. (2012). The value of Hydrophobicity coefficient was related to the large negative impact of the alkyl groups (Figure 3 and Table 3), which may have either a direct negative effect on plant growth or a role in reducing the positive bioactivity of polar molecules. In fact, hydrophilic bioactive molecules may become trapped within the hydrophobic domains of HLS, thus limiting their interactions with plant tissues and ultimately and significantly affecting HLS biological properties (Spaccini et al., 2002; Piccolo et al., 2019). In contrast to our results, García et al. (2016) reported a positive effect of alkyl C on plant development. This discrepancy may be attributed to specific differences in the types of alkyl functions between our materials and those of García et al. (2016). Moreover, it should be noted that the values of the regression coefficients based on the semi-quantitative ^13^C-CPMAS-NMR technique are also dependent on other X variables, since the values of predictors are inter-dependent and sum to 100%. For this reason, the regression coefficient for each X variable is strongly affected by the raw value of other predictors, since when an X value increases other(s) necessarily decreases. Hence, the other regression coefficients could actually account for the negative effect of alkyl moieties, the effect of which is then not reflected in the calculated regression coefficient for such chemical group. Besides the structural diversity of the various HS, it should be noted that differences in their biological activity could be related to the heterogeneous ways the bioactivity was assessed. Indeed, the biological effect of HS had been evaluated on different plants at different phenological states, grown in different conditions. Therefore, the lack of homogeneity in the employed bioassays may also explain the discrepancies between our results and those of the cited studies (Zandonadi et al., 2013).

In line with the PLS regression based on ^13^C-CPMAS-NMR spectra, the QSAR derived from ^31^P-NMR spectra indicated the positive effect of aliphatic OH groups, and suggested an important role of both guaiacyl and *p*-hydroxyphenyl units in modulating the HLS bioactivity (Figure 3 and Table 3). The positive biological effect of these compounds toward root development had already been described earlier and confirmed thereafter (DeKock and Vaughan, 1975; Kuiters, 1989; Reigosa et al., 1999). *p*-Coumaric, *p*-hydroxybenzoic, and vanillic acids, and *p*-vanillin were found to promote the root growth of six plant species in a dose-dependent manner. In particular, small concentrations stimulated root growth or were ineffective, whereas larger concentrations inhibited plant development (Kuiters, 1989; Reigosa et al., 1999). The positive effect of phenols on plant development may be due to their hormone-like activity in germination, as well as in root and coleoptile elongation. Savy et al. (2017a) showed the gibberellic-like effect of AD, as assessed by a specific bioassay. Pizzeghello et al. (2006) and Nardi et al. (2000) also reported the gibberellic-like effect of vanillin, and *p*-hydroxybenzoic and vanillic acids. Moreover, it was also found that a phenolic mixture from grape (*Vitis vinifera* L.) had a stimulating effect of on the activity of α-amylase, β-amylase, catalase and protease, which are enzymes known to be involved in the germination process (Tudose, 2002). Some of the listed phenolic compounds had been identified in our HLS, and might be responsible for their biological activity (Savy and Piccolo, 2014; Savy et al., 2015a, 2016a, 2017b).

Moreover, it should be kept in mind that the validity of our models may vary according to the HLS mode of application. If HLS are used as seed coaters, our models would likely accurately predict the HLS bioactivity. Instead, if HLS are applied to soil, some molecules may be selectively adsorbed onto clay surfaces or absorbed into pre-existing clay-humic complexes, hence significantly affecting HLS bioactivity (Xiao et al., 2019). Finally, the HLS may show a different bioactivity if sprayed on plant leaves rather than applied to the seeds, due the different interactions of such materials with the plant organs (Zandonadi et al., 2013; Sleighter et al., 2015).

### QSAR Derivation May Support in Designing the Next-Generation Biostimulants

Altogether, our findings point out that HLS bioactivity was positively affected by the presence of lignin-derived monomers, while hydrophobic, alkyl components and free carboxyl groups had a negative influence (Figure 3 and Table 3). Hence, the application of lignocellulosic biomasses containing large amount of guaiacyl and *p*-hydroxyphenyl units, such as herbaceous Angiosperms, can be recommended. Additionally, longer alkaline hydrolysis times may be adopted to reach a more extensive lignin depolymerization in order to increase the concentration of free phenolic units in HLS (Guizani and Lachenal, 2017), although a selective oxidation of lignin is difficult to achieve. In order to overcome undesired reactions and control the HLS oxidation, the implementation of specific oxidative strategies are required (Cha et al., 2017; Guo et al., 2018; Jeong et al., 2018). Finally, a removal of alkyl moieties from HLS may be envisaged to reduce the amount of such inhibitory compounds, for example by extracting them from the lignocellulosic matrix. However, because HLS bioactivity may be due to compounds bearing different chemical groups, further research should be carried out in order to implement analytical techniques for the isolation of the biologically active compounds present in the HLS (Nebbioso et al., 2014; Sleighter et al., 2015). Humeomics is an emerging protocol useful to progressively reduce the large chemical heterogeneity of HS and HLS (Nebbioso et al., 2014). Alternatively, preparative size exclusion chromatography can also be exploited to obtain fractions with similar hydrodynamic *radius* (Conte et al., 2007). Once the biological activity of HLS is understood, new biological trials should be carried out with the separated HLS fractions, and new, more robust PLS models could be obtained. These results may be then useful to select or modify HLS structure in order to fine-tune their bioactivity toward plant development.

## Conclusion

Several PLS models were created in order to derive a QSAR between maize early growth and HLS isolated from lignin-rich agro-industrial residues, based on their molecular characteristics evaluated by ^13^C-CPMAS-NMR spectra and by ^31^P-NMR spectroscopy after ^31^P-derivatization. The developed models explained more than 72% of the cumulative variance regardless of the employed predictors, and suggested the relevant positive role of aryl-containing molecules and *O*-alkyl groups of lignin origin on root and coleoptile elongation. Moreover, positive regression coefficients were calculated for both guaiacyl- and *p*-hydroxyphenyl –derived molecules. Conversely, the PLS regression indicated the negative role of alkyl groups and free carboxyl/esterified functions on plant development. Since our models were built to predict the bioactivity of HLS at the early growth stage of maize, they provided information on the chemical characteristics of lignin-derived HLS relevant for seedling establishment and the plant organs impacted by HLS application. Further studies are needed to relate HLS molecular structure to specific, biostimulant-related plant traits, such those relevant to nutrient use efficiency, tolerance to abiotic stress and/or crop quality, especially for plants at more advanced growth stages. Moreover, further research should be devoted to the implementation of separation techniques in order to attempt the isolation of the biologically active compounds present in the HLS. Once the biologically active molecules have been identified, new, more robust PLS regressions can be calculated. Then, the researchers may adopt chemical technologies to control the molecular composition of HLS with the aim to enhance the functions responsible for their bioactivity. Tailored-made HLS with the desired bioactivity toward plant physiology and development may be therefore developed, with the aim to exert efficient and sustainable biostimulant capacities to improve crop yields and their resilience to adverse environment.

## Data Availability Statement

All datasets generated for this study are included in the article/Supplementary Material.

## Author Contributions

DS conceived the manuscript, carried out the statistical analyses, and drafted the article. YB supervised the statistical tests and revised the manuscript. PD and VC contributed to the statistical analyses and revised the manuscript. PJ and AP revised the manuscript.

## Conflict of Interest

The authors declare that the research was conducted in the absence of any commercial or financial relationships that could be construed as a potential conflict of interest.

## Abbreviations

^13^C-CPMAS: ^13^C-cross polarization-magic angle spinning NMR spectroscopy
AD: humic-like substances isolated from giant reed
BYP 1 and BYP 2: humic-like substances isolated from two different biorefinery residues
CAR: humic-like substances isolated from cardoon
EUC: humic-like substances isolated from eucalypt
HLSs: humic-like substances
HS: humic substances
LIM: humic-like substances isolated from black poplar grown along the Limatola creek
MG: humic-like substances isolated from miscanthus
NMR: nuclear magnetic resonance
PLS: projection on latent structure
QSAR: quantitative structural-activity relationship
RIP: humic-like substances isolated from black poplar grown along the Ripiti creek
TMDP: 2-chloro-4,4,5,5-tetramethyl-1,3,2,-dioxaphospholane
VIP: variable importance plot.
