# Supplementary material for: Quantitative Structure-Activity Relationship of Humic-Like Biostimulants Derived From Agro-Industrial Byproducts and Energy Crops
Source: Front Plant Sci. 2020 May 26;11:581. doi: 10.3389/fpls.2020.00581 (PMC7264396; doi:10.3389/fpls.2020.00581)
Supplement: Supplementary file 1 [file Data_Sheet_1.docx]

**SUPPORTING MATERIAL**

# Quantitative Structure-Activity Relationship of humic-like biostimulants derived from agro-industrial byproducts and energy crops

Davide Savy^1*^, Yves Brostaux^2^, Vincenza Cozzolino^3,4^, Pierre Delaplace^1^, Patrick du Jardin^1^, Alessandro Piccolo^3,4^

^1^ Plant Sciences, Gembloux Agro-Bio Tech, University of Liège, 2 Passage des Déportés, B-5030 Gembloux, Belgium

^2^ Statistical modelling and development, Gembloux Agro-Bio Tech, University of Liège, 2 Passage des Déportés, B-5030 Gembloux, Belgium

^3^ Interdepartmental Research Centre of Nuclear Magnetic Resonance for the Environment, Agri-Food and New Materials (CERMANU) - University of Naples Federico II, Via Università 100, 80055 Portici, Italy.

^4^ Department of Agricultural Sciences, Università di Napoli Federico II, Via Università 100, 80055 Portici, Italy

*** Corresponding author. E-mail address: davide.savy@unina.it**

Pages: 9

Supporting Figures: 6

**Figure Captions**

**SUPPORTING FIGURE SF1** ^13^C-Cross Polarization Magic Angle Spinning (CPMAS) NMR spectra of Humic-Like Substances isolated form giant reed (AD), miscanthus (MG), cardoon (CAR), eucalypt (EUC), poplar grown along the Ripiti (RIP) Limatola creeks (LIM), and two biorefinery residues (BYP 1 and BYP 2) (adapted from Savy and Piccolo, 2014; Savy et al., 2015a; 2017a).

**SUPPORTING FIGURE SF2** ^31^P-NMR spectra of ^31^P-derivatized Humic-Like Substances isolated form giant reed (AD), miscanthus (MG), cardoon (CAR), eucalypt (EUC), poplar grown along the Ripiti (RIP) Limatola creeks (LIM), and two biorefinery residues (BYP 1 and BYP 2) (adapted from Savy et al., 2015a; 2016; 2017a).

**SUPPORTING FIGURE SF3** Lignin monomeric units: *p*-hydroxyphenyl (P), guaiacyl (3-methoxy-4-hydroxyphenyl-G) and syringyl (3-methoxy-4-hydroxyphenyl-S).

**SUPPORTING FIGURE SF4** Score plot and loading plot for the first two latent components for the elongation of total root *apparatus* (A, C) and coleoptile (B, D) as derived by exploiting ^31^P-NMR-derived results.

**SUPPORTING FIGURE SF5** Predicted VS actual (observed) plot for the elongation of primary root (PR), lateral seminal root (LSR), total root (TRL) and coleoptile (Col) as derived by exploiting ^13^C-CPMAS-NMR-derived results.

**SUPPORTING FIGURE SF6** Predicted VS actual (observed) plot for the elongation of primary root (PR), lateral seminal root (LSR), total root (TRL) and coleoptile (Col) as derived by exploiting ^31^P-NMR-derived results.

**
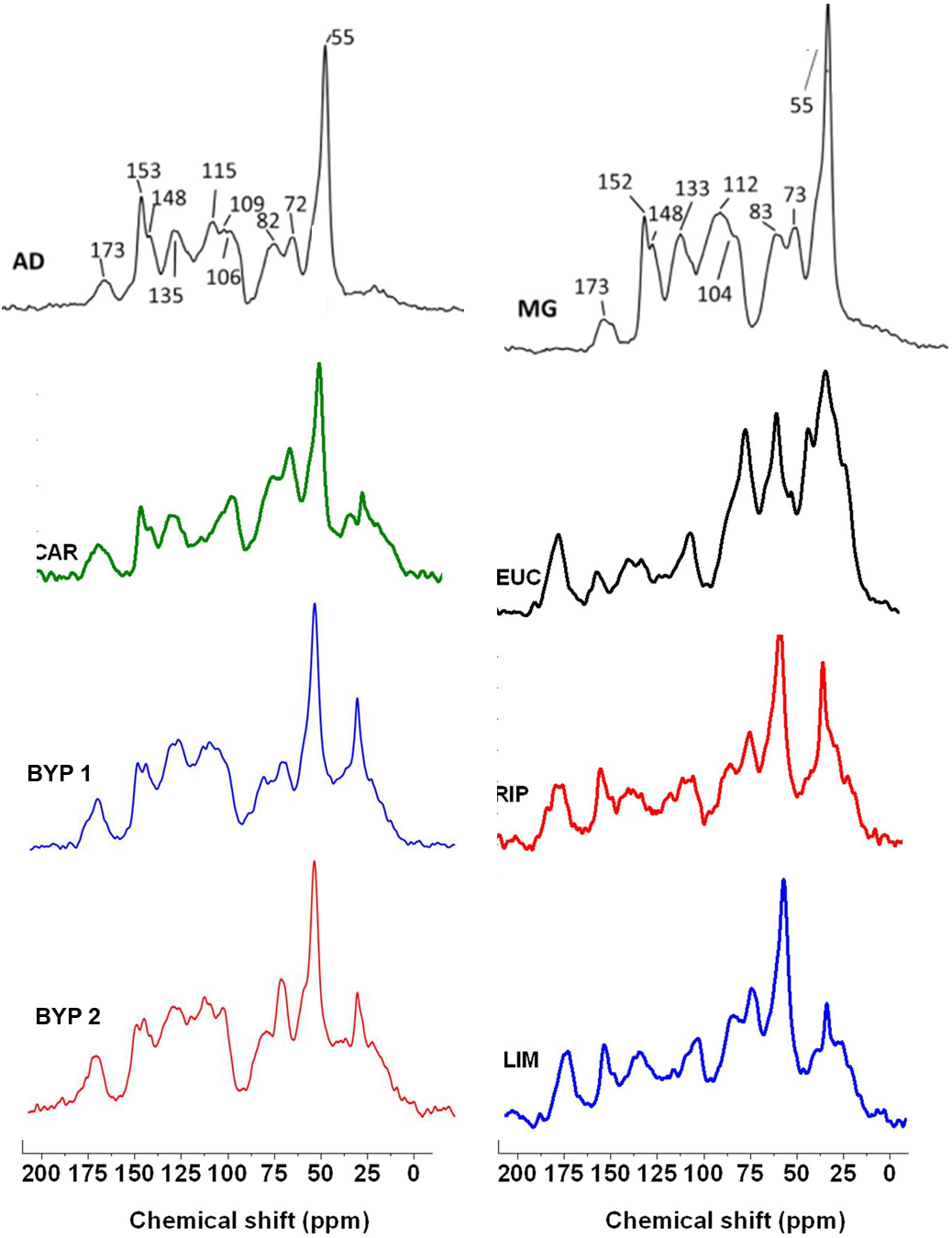
**

# SUPPORTING FIGURE SF1

**
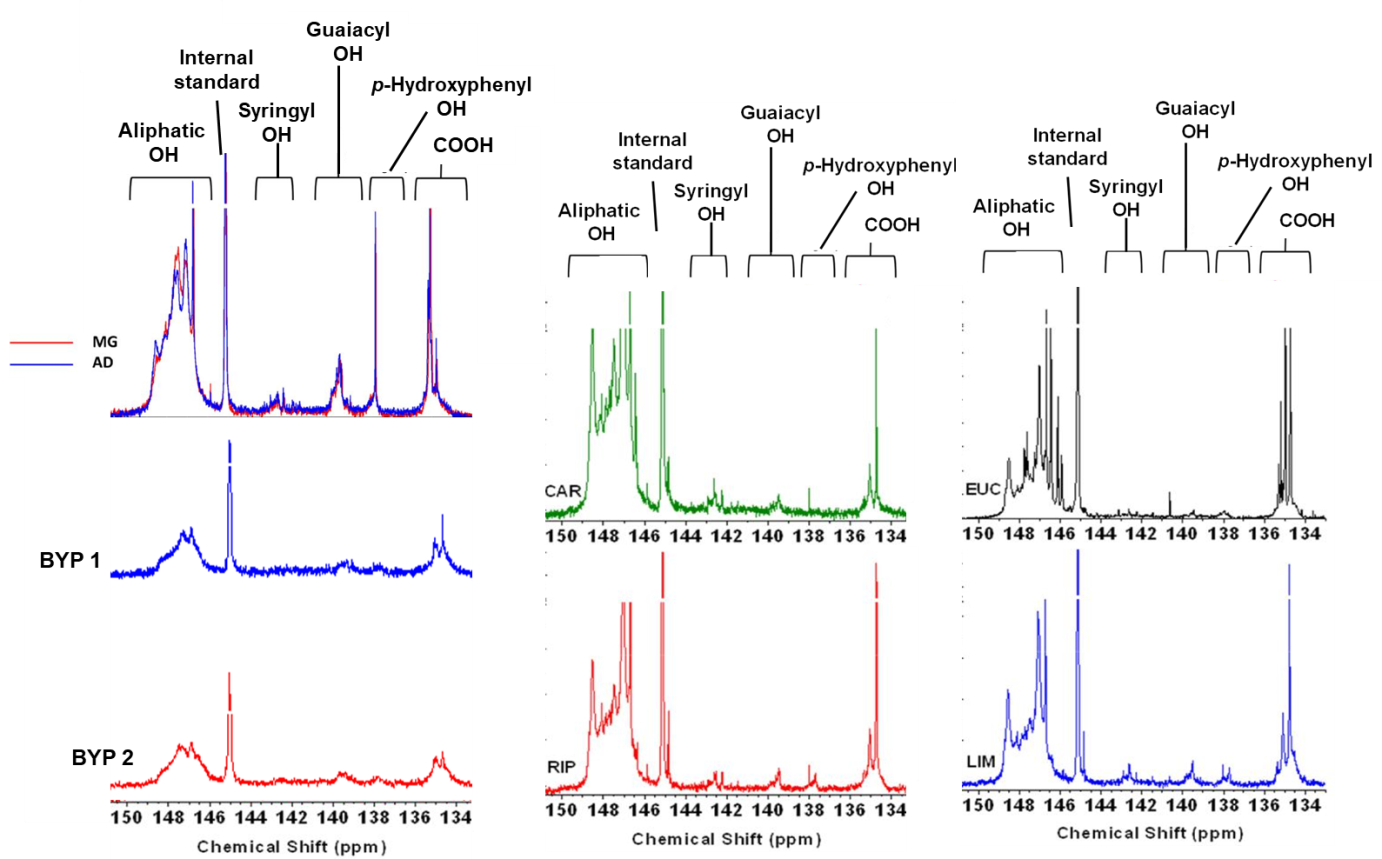
**

# SUPPORTING FIGURE SF2

**
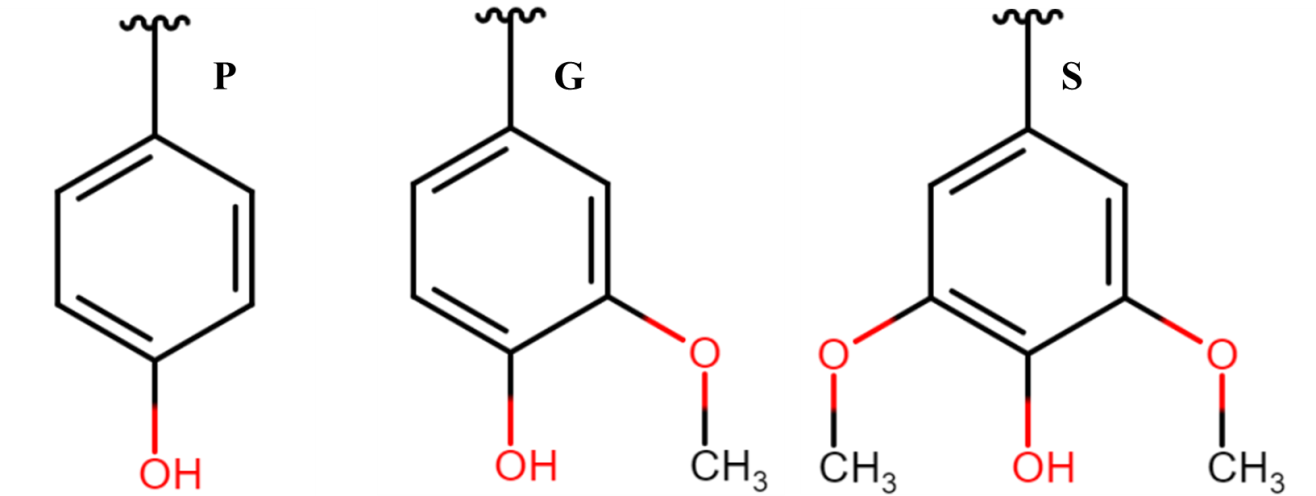
**

# SUPPORTING FIGURE SF3

**
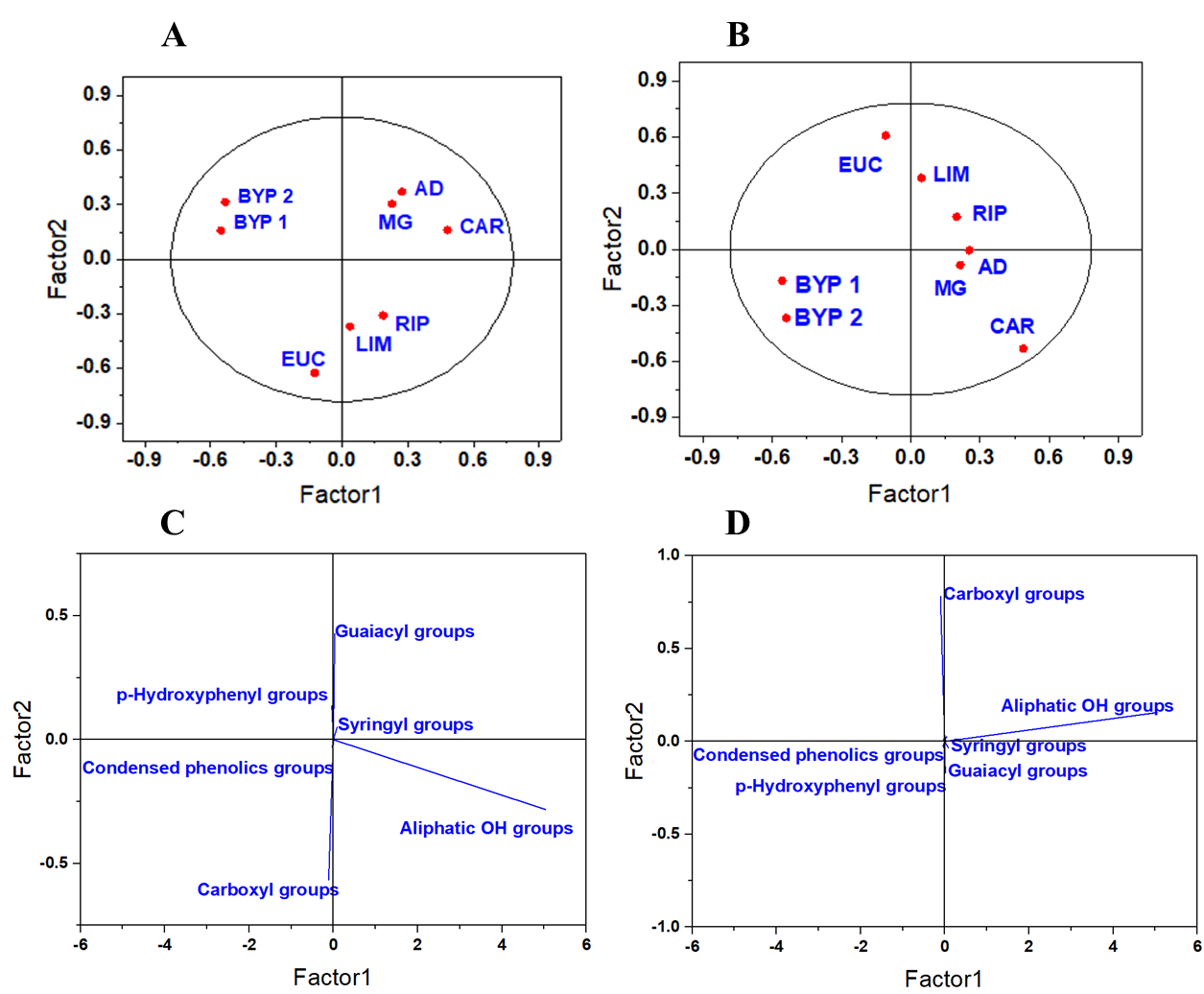
**

# SUPPORTING FIGURE SF4


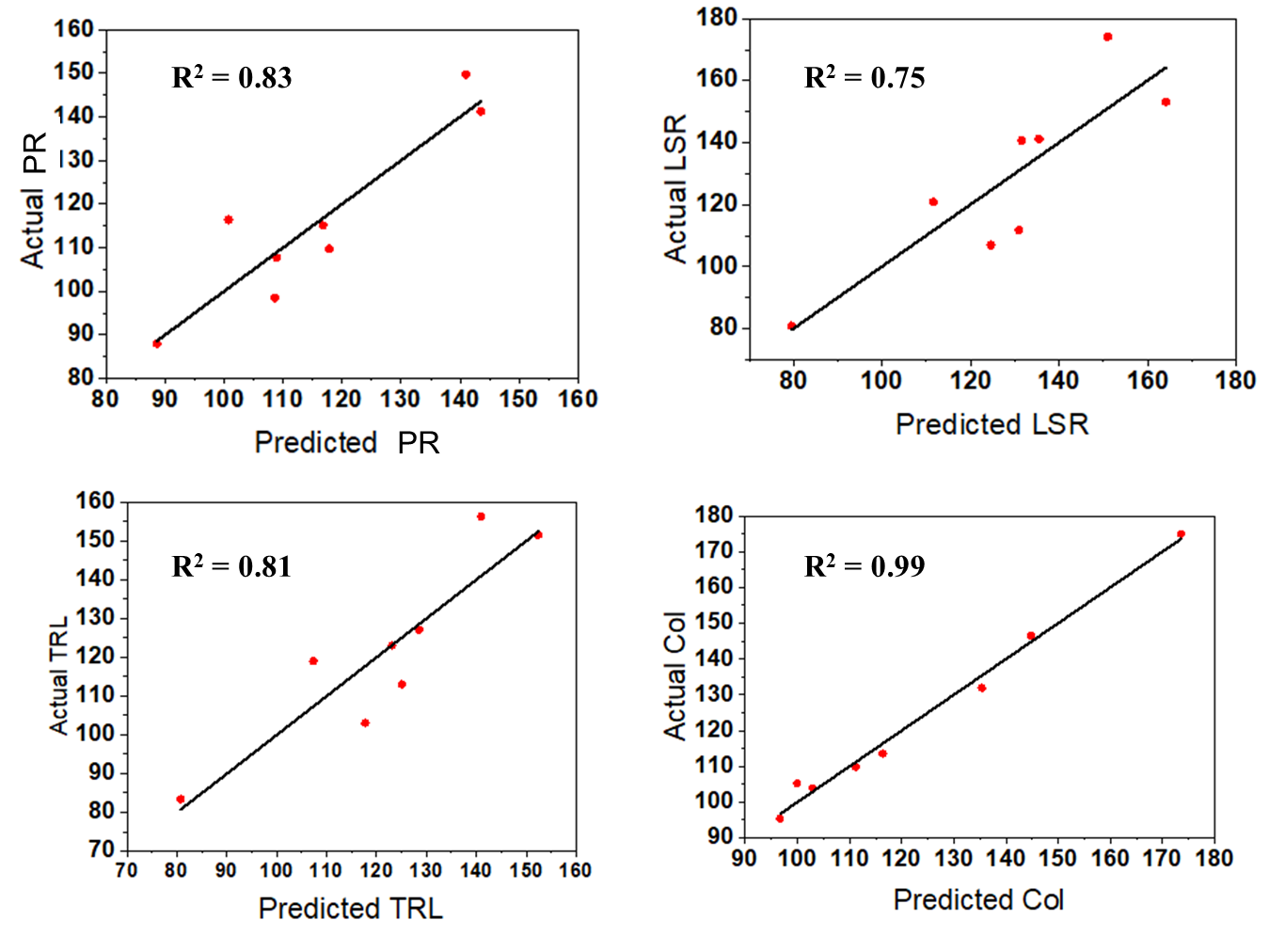


# SUPPORTING FIGURE SF5

**
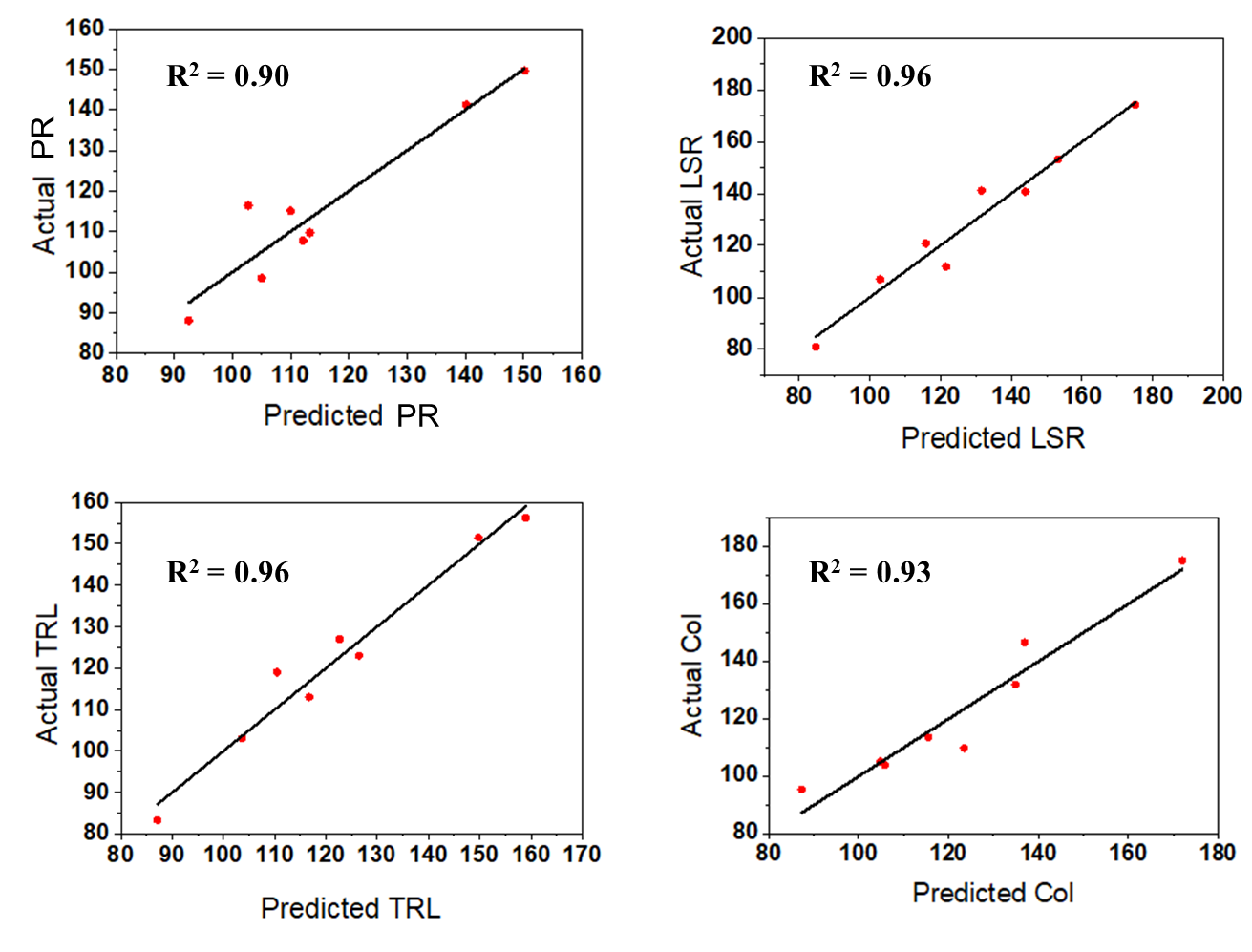
**

# SUPPORTING FIGURE SF6
